# Sufficient virus-neutralizing antibody in the central nerve system improves the survival of rabid rats

**DOI:** 10.1186/1423-0127-19-61

**Published:** 2012-06-26

**Authors:** Pi-Hung Liao, Hui-Hua Yang, Ping-Tse Chou, Ming-Hseng Wang, Po-Chun Chu, Hao-Li Liu, Li-Kuang Chen

**Affiliations:** 1 Institute of Medical Sciences, Tzu Chi University, Hualien, Taiwan; 2 Department of Emerging Infectious Pathogen Research Laboratory, Buddhist Tzu Chi General Hospital, Hualien, Taiwan; 3 Department of Life Science, Tzu Chi University, Hualien, Taiwan; 4 Department of Electrical Engineering, Chang-Gung University, Taoyuan, Taiwan; 5 Department of Laboratory Diagnostics, Medical School, Tzu Chi University, Hualien, Taiwan; 6 Department of Clinical Pathology, Buddhist Tzu Chi General Hospital, Hualien, Taiwan; 7 Department of Clinical Pathology, Buddhist Tzu Chi General Hospital, Medical School, Tzu Chi University, No 707, Section 3, Chung-Yang Road, Hualien, 970, Taiwan

**Keywords:** Rabies, Blood–brain barrier, Central nerve system, Cerebrospinal fluid, Occludin, Hypertonic breakdown, Virus-neutralizing monoclonal antibody, Passive immunotherapy

## Abstract

**Background:**

Rabies is known to be lethal in human. Treatment with passive immunity for the rabies is effective only when the patients have not shown the central nerve system (CNS) signs. The blood–brain barrier (BBB) is a complex functional barrier that may compromise the therapeutic development in neurological diseases. The goal of this study is to determine the change of BBB integrity and to assess the therapeutic possibility of enhancing BBB permeability combined with passive immunity in the late stage of rabies virus infection.

**Methods:**

The integrity of BBB permeability in rats was measured by quantitative ELISA for total IgG and albumin levels in the cerebrospinal fluid (CSF) and by exogenously applying Evans blue as a tracer. Western blotting of occludin and ZO-1, two tight junction proteins, was used to assess the molecular change of BBB structure.

The breakdown of BBB with hypertonic arabinose, recombinant tumor necrosis factor-alpha (rTNF-γ), and focused ultrasound (FUS) were used to compare the extent of BBB disruption with rabies virus infection. Specific humoral immunity was analyzed by immunofluorescent assay and rapid fluorescent focus inhibition test. Virus-neutralizing monoclonal antibody (mAb) 8-10E was administered to rats with hypertonic breakdown of BBB as a passive immunotherapy to prevent the death from rabies.

**Results:**

The BBB permeability was altered on day 7 post-infection. Increased BBB permeability induced by rabies virus infection was observed primarily in the cerebellum and spinal cord. Occludin was significantly decreased in both the cerebral cortex and cerebellum. The rabies virus-specific antibody was not strongly elicited even in the presence of clinical signs. Disruption of BBB had no direct association with the lethal outcome of rabies. Passive immunotherapy with virus-neutralizing mAb 8-10E with the hypertonic breakdown of BBB prolonged the survival of rabies virus-infected rats.

**Conclusions:**

We demonstrated that the BBB permeability was altered in a rat model with rabies virus inoculation. Delivery of neutralizing mAb to the infected site in brain combined with effective breakdown of BBB could be an aggressive but feasible therapeutic mode in rabies when the CNS infection has been established.

## Background

Rabies is a highly lethal disease caused by a neurotrophic rabies virus. It has been estimated that about 55,000 persons died from rabies each year. In spite of the prevalence occurred primarily in Africa and Asia, the disease exists globally [1]. Vaccines have been well developed for the prophylaxis of the disease. When individuals are infected with rabies, early post-exposure prophylaxis (PEP) treatment may avoid death. Unfortunately, the PEP treatment is deemed ineffective once the clinical signs have appeared [2,3]. The mortality is almost 100% once clinical signs were observed, although few cases can survive successfully after the onset of symptoms [4-8]. Because specialized blood–brain barrier (BBB) can protect the CNS from a variety of injuries, it is reasonable to assume that exclusion of immune cells or mediators from entering the CNS may lead to a lethal outcome.

Recently, it had been reported that the BBB was more permeable in mice infected with laboratory-attenuated CVS-F3 than mice infected with silver-haired bat rabies virus (SHBRV) [9,10]. The survival of SHBRV infected mice was improved by enhancing the inflammatory response and the delivery of immune effectors into the CNS [9,10]. These studies suggested that the failure of increasing BBB permeability may promote the disease development in the pathogenic rabies virus-infected mice. Nevertheless, increasing levels of total protein, blood cells count and rabies virus-specific immunoglobulin in the CSF of patients infected with pathogenic rabies virus have been reported [7,8,11-13]. Neuroimaging showed abnormality of brainstem, thalamus, hypothalamus, hippocampus and basal ganglia, and subcortical and deep white matter in some patients diagnosed with rabies [12,14,15]. These clinical findings implied the possibility of the BBB dysfunction in human rabies. Whether alterations in the BBB permeability could merely exist in attenuated but not pathogenic strain or enhancement of the BBB permeability could prevent disease progression after rabies virus infection is not completely understood.

In the present study, we determined the BBB permeability and assessed the correlation between BBB integrity and fatality in a rat model infected with pathogenic rabies virus isolated from a dog-bite patient. Further, we used passive immunotherapy with virus-neutralizing mAb with BBB disruption to assess the practicability of this therapeutic strategy in the late stage of rabies.

## Methods

### Animals, virus and mAb treatment

Eight- to ten-week-old male LEW/SsNNarl (LEW) rats were purchased from National Laboratory Animal Center, Taipei, Taiwan. They were infected via gastrocnemius muscle inoculation with 2 × 10^6^ f.f.u. (focus-forming units) rabies virus (Genotype 1; Accession AY431027, originally from a rabid dog) in 100 μl phosphate buffer saline (PBS); control animals received the same volume of PBS. The animals were monitored twice daily and euthanized by intraperitoneal injection of pentobarbital (150 mg/kg). In passive immunity studies, the rats were injected with rabies virus glycoprotein-specific, neutralizing mAb 8-10E (IgG1κ, 1,800 IU/ml, generated at our laboratory) or with normal saline alone at the indicated time. All procedures were carried out according to the protocols approved by the Institutional Animal Care and Use Committee of Tzu-Chi University.

### Serial collection of CSF from rats

The surgery was performed according to pervious studies with minor modifications [16,17]. Briefly, rats were anaesthetized with ketamine (80–100 mg/kg) and xylazine (5–10 mg/kg) and immobilized on a stereotaxic frame (model 51600, Stoelting, Wood Dale, IL, USA) with self-made swivel mounts (15 cm). The skin overlying the occipital bone was incised and the underlying tissue was prepared as such that the atlanto-occipital membrane in between the occipital bone and the upper cervical vertebral was exposed. The cannula was made of polyethylene tubing (PE-10). A hole was drilled through the occipital bone, and the tube was allowed to enter the cisterna magna. The tubing was occluded with a blunt 27 G needle to prevent the escaping of the CSF. The cannula was further attached to the skull using dental cement (Lang Dental MFG, Chicago, IL, USA). The CSF samples (25–50 μl) were collected and stored at −80°C until analysis.

### Quantitative ELISA

Immunoglobulin G (IgG) and albumin in the CSF and serum were quantitated by sandwich ELISA using commercial kits (Bethyl Laboratories, Mongomery, TX, USA). Total IgG and albumin concentrations were measured in duplicate.

### Evans blue staining

BBB permeability was quantitatively evaluated by measuring the amount of extravasated Evans blue (EB, Sigma, St. Louis, MO, USA). EB (2%, 4 ml/kg body) was injected intravenously and allowed to circulate for 1 h [18]. After washing with normal saline, the brains were removed, weighted, and homogenized in 50% trichloroacetic acid and centrifuged for 20 min at 13,000 rpm (5415R, Eppendorf, Hamburg, Germany). The absorption of the supernatant was measured at 620 nm with a spectrophotometer (DU-7400, Beckman Coulter, CA, USA). The concentration of EB dye was calculated using a standard curve.

### Western blotting analysis

Conventional Western blotting was used. Briefly, brain samples were homogenized in ice-cold radioimmunoprecipitation (RIPA) buffer, supplemented with protease inhibitor cocktail tablets (Roche Diagnostics GmbH, Germany). The homogenates were centrifuged and protein concentrations of the supernatants were determined using a Pierce protein assay kit (Pierce, Rockford, IL, USA). Equal amounts of protein (80 μg) were loaded onto 7.5% or 10% sodium dodecyl sulfate-polyacylamide gel, transferred onto polyvinylidene difluoride membranes (Millipore, Bedford, MA, USA). The membrane was then blocked with 5% nonfat milk and incubated with primary antibodies overnight at 4°C. The primary antibodies and concentrations used were as follows: rabbit polyclonal anti-occludin (Zymed Laboratories, South San Francisco, CA, USA, 1:200), rabbit polyclonal anti-ZO-1 (Zymed Laboratories, USA, 1:200), and mouse monoclonal anti-β-actin (BD Biosciences, San Jose, CA, USA, 1:5000). Following washing and incubation with the secondary antibody (Chemicon, Temecula, CA, USA, 1:2000) for 60 min at room temperature, the membranes were then probed with chemiluminescence reagents using a commercially available kit (ECL Plus Western blotting detection system, Amersham Biosciences, Little Chalfont, Buckinghamshire, UK) to visualize the signals, followed by exposure to X-ray films (Kodak, Rochester, NY, USA). Intensities of the band were quantified with a densitometric analysis system (GS-800 Calibrated Densitometer, Bio-Rad, Hercules, CA, USA), and calculated as the optical density x area of band.

### Disruption of BBB permeability

Three methods were use to disrupt the BBB permeability. Hyperosmotic solution and TNF-α were performed as described previously [19-21]. Briefly, rats were anesthetized with ketamine (80–100 mg/kg) and xylazine (5–10 mg/kg), and infused via carotid artery with 1.6 M arabinose (0.12 ml/sec, constant for 30 sec) or 10^6^ IU human recombinant TNF-α protein (Peprotech Inc, Rocky Hill, NJ, USA). FUS treatment was described previously [22,23]. SonoVue® SF6-coated ultrasound microbubbles (2–5 mm mean diameter, 1.5 mg/kg; Bracco Diagnostics Inc., Princeton, NJ, USA) were administered intravenously by continuous injection with 0.2 ml saline solution containing 0.1 ml heparin by a micropump (0.6 ml/min). Moderate ultrasound power (4.3 W, equivalent to a pressure of 0.4 MPa) was delivered to the brain with the center of the focal zone positioned at a penetration depth of 2–3 mm. In order to increase the BBB permeability of entire half brain, multiple exposures were carried out to completely cover the hemisphere. Animals typically underwent 3 sonications, with the spacing between individual adjacent focal positions set to 3 mm. In each sonication, burst mode ultrasound was delivered with the following parameters: burst length = 10 ms, pulse repetitive frequency = 2 Hz, and sonication duration = 1 min.

### Measurement of serum and CSF antibody titers

Levels of rabies virus-specific total IgG from rats infected with rabies virus as well as uninfected control groups were measured by indirect immunofluorescent assay (IFA). The titer was defined as the reciprocal of the highest dilution factor of test samples in which 50% or more of the fields examined contained specifically fluorescing cells. Virus-neutralizing antibody (VNA) titers were determined by the rapid fluorescent focus inhibition test (RFFIT) as previously described [24,25]. The VNA titer was determined as the last dilution of serum that was capable of reducing the number of FFU by 50%.

### Passive immunotherapy with BBB opening

Experiments were divided into two parts: (1) for the passive immunotherapy alone, the rabies virus-infected rats were intraperitoneally administrated 10 mg mAb 8-10E, and (2) for the passive immunotherapy combined with BBB opening (BBBO), mAb 8-10E was administered 6 hrs before BBB disruption. The studies were carried out on day 7 and day 9 post-infection (p.i.). The rats were continually observed to day 40 p.i. and then euthanized for analysis.

### Reverse transcription-polymerase chain reaction (RT-PCR) analysis

Total RNA was extracted from the cerebral cortex, cerebellum, and spinal cord of rabies virus infected and control rats using the RNeasy Mini kit (Qiagen, Hilden, Germany) according to the manufacturer’s instructions. The nucleoprotein (N) gene of rabies virus was amplified by one-step RT-PCR. The nucleotide sequences of the primers used for PCR are forward primer, 5'- CTACAATGGATGCCGAC-3' and reverse primer, 5'-TCATAACGGAGAGATCGCCAC-3'. RT-PCR was performed with 4 μl of RNA (0.2-0.4 μg) and the OneStep RT-PCR kit (Qiagen, Germany) according to the manufacture’s instructions. Each reaction mixture was incubated at 50°C for 30 min, followed by 40 cycles at 94°C for 30 sec, 55°C for 30 sec, 72°C for 1 min, and 72°C for 10 min. Equal amounts of amplified PCR products were electrophoresed in 1.5% agarose gels containing ethidium bromide. The bands were visualized under UV light and photographed.

### Statistical analysis

All data were expressed as mean ± SEM. One-way ANOVA was used to compare means between experimental groups. Student’s *t*-test was used to determine the significance between groups. The survival rate was analyzed by the Kaplan–Meier method and log-rank statistics were used to test the difference between groups. The GraphPad Prism statistical package, version 5.0c (GraphPad Software, Inc., San Diego, CA, USA) was used for comparison. Differences were considered statistically significant at *p* < 0.05.

## Results

### Rabies viral antigens were detected in the CNS of LEW rats after challenge

Clinical signs of rabies infection appeared on day 9–10 p.i.. Unsteady gait and hunched back were observed in the beginning, swimming movement, slow motion, and limbs paralysis in the late stage. Rats died from the infection within 2 to 4 days once the neural signs were observed. The viral genome was detected by IFA, RT-PCR and immunohistochemistry on day 5 p.i. in the brain and spread out within the next 2 days (data not shown).

### BBB permeability was disrupted after rabies virus challenge

**Figure 1:**
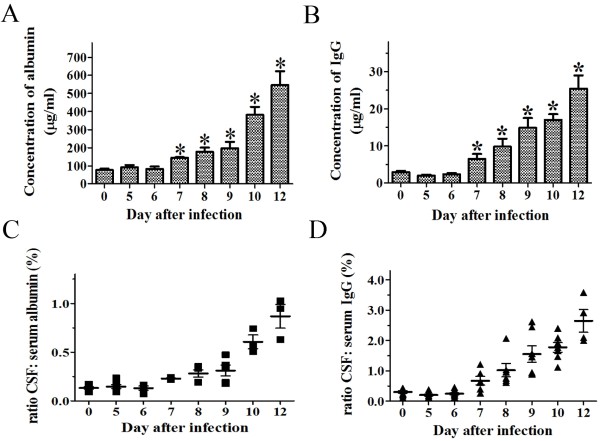
**BBB permeability was altered in the CSF of rats infected with rabies virus.** BBB permeability was assessed by measuring **(A)** albumin and **(B)** total IgG from the CSF after rabies virus infection and expressed as the concentration (μg/ml) (n = 8). Statistical significance of differences in permeability was calculated using Student’s *t*-test and are denoted by the symbol *p < 0.05. The CSF to serum ratios were presented in **(C)** for albumin and **(D)** for IgG. Each dot represented one individual rat.

To determine the possible alteration of BBB permeability, the CSF were serially sampled and analyzed. The status of the BBB permeability was monitored post-infection every single day by quantifying the albumin and total IgG in the CSF using quantitative ELISA. The results showed that the albumin and total IgG levels were elevated significantly on day 7 and continually elevated to day 12 p.i. (Figure 1A, B). The time course of the changes of the CSF to serum ratios of albumin and total IgG in rabies virus-infected rats is shown in Figure 1C and D. There were about 7- and 10-fold increase in albumin and total IgG, respectively, in the CSF on day 12 p.i. when compared to uninfected rats. The time course of the increase in BBB permeability paralleled that of animal illness.

### BBB permeability alterations were observed in the cerebellum and spinal cord with EB staining

**Figure 2:**
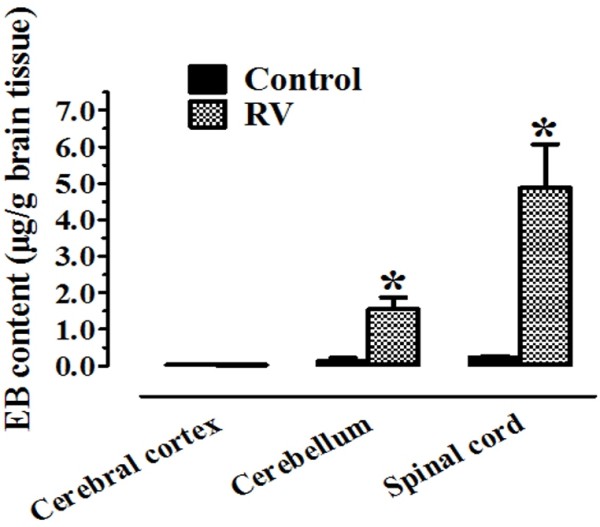
**Localization of BBB permeability changes in the brain of rabies virus-infected rats.** Brains were removed from Evans blue-treated rats either in the control or rabies virus-infected animals on day 10 to 11 p.i.. BBB permeability was assessed by measuring the EB content which was determined by a standard curve. Compared with controls (n = 4), rabies virus-infected rats (n = 8) showed a significant increased in the amount of EB (*p < 0.05).

To evaluate the regional differences in BBB permeability alterations, we measured the extravasations of EB from the circulation into the CNS tissues on day 10 to 11 p.i.. Staining of Evans blue was found mainly in the cerebellum and spinal cord. There were about 11- and 20-fold increase in the cerebellum and spinal cord, respectively, compared to control rats (Figure 2). In contrast, relatively mild cerebral cortical BBB breakdown was observed as revealed by a no significant difference in EB staining. The results indicated that the integrity of the BBB was changed after rabies virus infection, and the major alteration were observed in the cerebellum and spinal cord.

### Tight junction protein occludin was degraded after rabies virus infection

**Figure 3:**
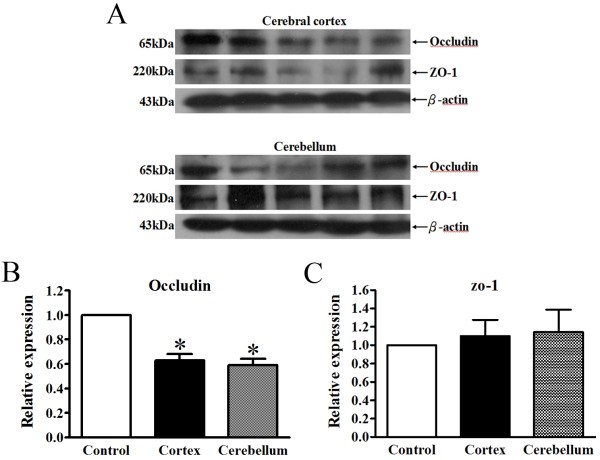
**Expression of tight junction protein, occludin, was decreased in the brains of rabies-infected rats.** Western blotting analyses of tight junction proteins, occludin and ZO-1, were shown in **(A)** the cerebral cortex and cerebellum. Relative levels of tight junction protein **(B)** occludin and **(C)** ZO-1 expression were determined by densitometric analysis. All samples analyzed (n = 4) were normalized to the intensity of corresponding β-actin bands. Results are expressed as mean ± SEM. *p < 0.05, significantly different from the control rats.

To investigate whether BBB breakdown in the brain is associated with the changes of endothelial tight junction proteins, occludin and ZO-1, from the cerebral cortex and cerebellum were immunoblotted. Figure 3A shows that the expression levels of occludin in the cerebral cortex and cerebellum were decreased after rabies virus infection. Occludin levels in the cerebral cortex and cerebellum were decreased by 37 ± 9% and 41 ± 9%, respectively (Figure 3B). Conversely, protein levels of ZO-1 were not significantly changed in the cerebral cortex and cerebellum (Figure 3C). These data indicated that rabies virus infection can disrupt BBB permeability through down-regulating the expression of occludin but not ZO-1 in the brain.

### The extent of BBB opening via hypertonic solution, TNF-α or FUS treatment

**Figure 4:**
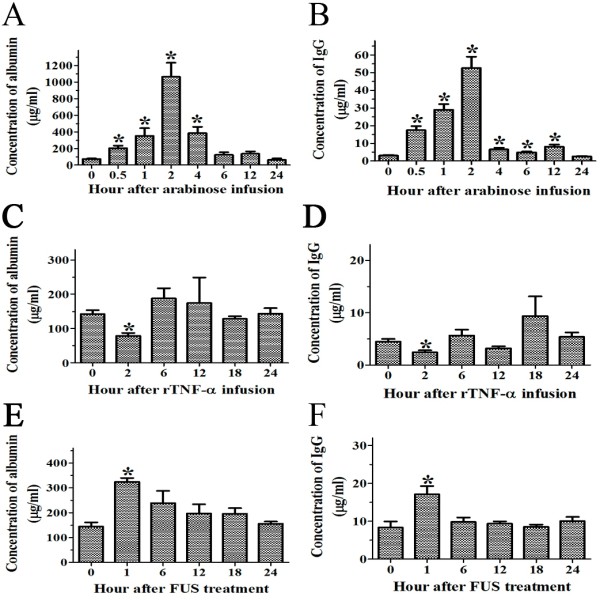
**Alteration of BBB permeability was compared in three methods.** BBB permeability was assessed by measuring the albumin **(A, C, E)** and total IgG **(B, D, F)** contents from the CSF after hypertonic arabinose (n = 8), rTNF-α (n = 5) and FUS (n = 5) treatment. The data were expressed as the concentration (μg/ml). Statistical significance of differences in BBB permeability was calculated using Student’s *t*-test and are denoted by the symbol *p < 0.05.

To measure the extent of BBB opening after hypertonic arabinose, TNF-α or FUS treatment, the albumin and total IgG levels of the CSF were continually analyzed using quantitative ELISA. In hypertonic breakdown with arabinose, the albumin and total IgG levels were elevated within 0.5 hr and reached the peak at 2 hr. The highest concentration was 1062.38 ± 146.10 μg/ml for the albumin and 52.59 ± 5.22 μg/ml for the total IgG. After 4 and 12 hr, the albumin and total IgG returned gradually to the control levels (Figure 4A, B). Following rTNF-α infusion, the BBB permeability was significantly decreased only at 2 hr; no significant effect was observed at other time points (Figure 4C, D). After FUS treatment, the BBB permeability was increased significantly at 1 hr and returned to control levels at 6 hr. The concentration of albumin was 324.40 ± 15.61 μg/ml and total IgG was 17.11 ± 2.17 μg/ml at 1 hr post treatment (Figure 4E, F).

### BBB opening was not the primary determinant for lethality of rabies virus infection

**Figure 5:**
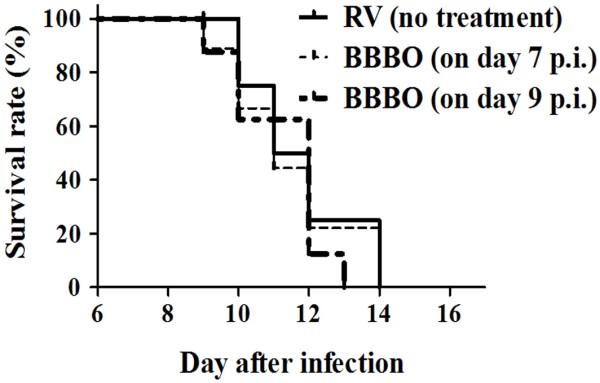
**Survival percentage of rabies virus-infected rats infused with or without BBBO.** Groups of rats (n = 7) after rabies virus (RV) infection were infused with or without hyperosmotic arabinose to disrupt BBB on day 7 and 9 p.i. and assessed the morbidity and mortality. BBBO was performed as described in the Methods. Comparison between RV alone and RV plus BBBO showed no significant difference between each groups (*p* > 0.05).

Our study showed that BBB opening (BBBO) was significant elevated following hypertonic arabinose infusion in rats. The enhancement of BBB permeability with hypertonic arabinose was performed on day 7 and 9 p.i.. The rats treated with or without hyperosmotic solution all died after rabies virus infection. Figure 5 showed that the percentage of survival was not different between the control (infection only) and the experimental groups (infection + BBBO). It could be postulated that the enhancement of BBB permeability was not the primary determinant for lethality of rabies virus infection.

### Specific anti-rabies virus antibody of the CNS tissue was not detected in the CSF and was lower in peripheral sera

**Figure 6:**
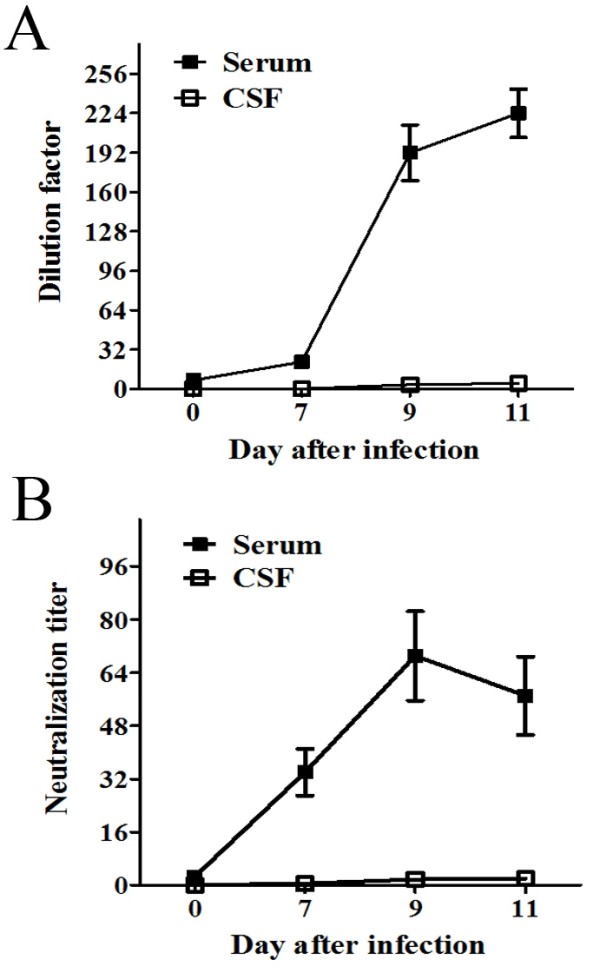
**Rabies virus-specific antibody productions were slightly elicited after infection.** Kinetics of **(A)** specific IgG and **(B)** virus-neutralizing antibody (VNA) production in the sera and CSF of rabies virus-infected rats (n = 8) were assessed by IFA and RFFIT, respectively. Levels of antibodies are presented as the geometric mean ± SEM of the last dilution of serum and CSF.

To estimate the humoral immunity of the rabies virus-infected rats, we measured the rabies virus-specific IgG and virus-neutralizing antibody (VNA) titer in the serum and CSF using IFA and RFFIT. The IFA results showed that the rabies virus-specific antibody was detected in the sera, but the mean of dilution factor was lower than 1:256 on day 11 p.i.. In the CSF, the antibody level was very low and the mean of dilution factor was less than 1:8 (Figure 6A). Similarly, the RFFIT also showed that the rabies virus-neutralizing titer was slightly increased in the sera and was limited in the CSF (Figure 6B). Consequently, the virus-specific antibody and immune response were insufficient to protect the animals from rabies virus infection.

### Rabid rats survived longer when the virus-neutralizing mAb entered the CNS

**Figure 7:**
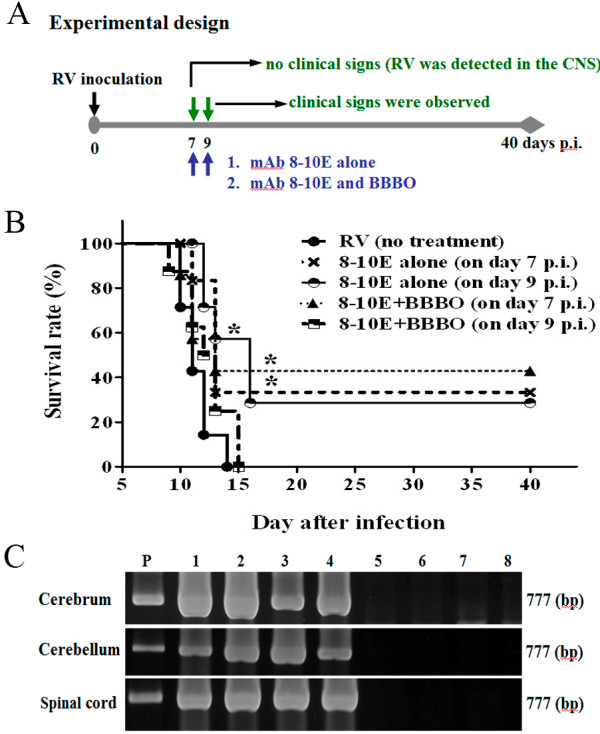
**Survival of rabies virus-infected rats treated with passive immunotherapy alone or in combination with BBBO. (A)** Diagram of treatment schedule: on day 7 and 9 p.i., group 1 animals received mAb 8-10E alone (n = 7) and group 2 animals received mAb 8-10E + BBBO (n = 8), **(B)** Survival of rats following infection with four different treatment: (1) 8-10E alone on day 7 p.i., (2) 8-10E + BBBO on day 7 p.i., (3) 8-10E alone on day 9 p.i., and (4) 8-10E + BBBO on day 9 p.i.. Rats were monitored for morbidity and mortality for 40 days. 8-10E: indicated that rats was injected with virus-neutralizing mAb 8-10E. BBBO: indicated that the BBB permeability was enhanced with hypertonic arabinose infusion. Statistical significance of differences in survival rats was denoted by the symbol *p<0.05. **(C)** Detection of viral RNA in the brain tissues infected with rabies virus. Viral nucleoprotein mRNA expression was detected in the cerebrum, cerebellum and spinal cord of rats died of BBBO and mAb 8-10E + BBBO treatment on day 14 p.i. (numbered 1, 2, 3, 4). The size of the amplified fragment was estimated to be 777 bp. No viral genome was detected in the brain tissues and spinal cord of rats survived for 40 day p.i. (numbered 5, 6, 7, 8). The lane marked “P” is the positive control taken from a cultured virus suspension.

To examine further if the survival rate may be increased when sufficient mAb crosses into the CNS, 10 mg rabies virus neutralizing mAb 8-10E (9000 IU) was administered to rabies virus-infected rats with or without BBBO (Figure 7A). Administration of mAb 8-10E alone on day 7 p.i. increased the survival rate from 0 to 33.3% and up to 42.9% when mAb 8-10E was combined with BBBO. Treatment with mAb alone on day 9 p.i. also increased the survival percentage to 28.6%. However, there was no further improvement in rats treated with mAb 8-10E combined with BBBO on day 9 p.i.. Instead, all the rats expired within 15 days p.i. (Figure 7B). Approximately half of the rats which were administered mAb 8-10E with BBBO on day 7 p.i. survived at least for forty days. There was a significant difference between 8-10E alone (on day 7 p.i.), 8-10E ± BBBO (on day 7 p.i.), and 8-10E alone (on day 9 p.i.) vs. control in the survival analysis. To clarify the CNS infectivity after BBBO and mAb treatment, rabies viral genome was amplified by RT-PCR. The viral genome in cerebrum, cerebellum and spinal cord was not detectable from the control and those groups of rats survived to day 40 p.i. (Figure 7C). In contrast, viral genome was found in the brain tissues from rats died of severe neurological signs (Figure 7C).

## Discussion

The BBB is a functional and structural component of the CNS vasculature that serves important protective functions for the CNS. Many clinical symptoms, immune responses, pathological changes and disease outcome in neurodegeneration or infectious diseases have been associated with the BBB permeability [26]. Alterations of BBB permeability have been reported after pathogenic rabies virus infection in human. Elevation of immune cells and leakage of serum components from circulation could be detected in some patients suffered from rabies [7,8,11-13]. In the present study, we demonstrated that the BBB permeability was altered after pathogenic rabies virus infection in a rat model, which was not consistent with previous studies in mice [9,10]. It could be speculated that in some animals, like laboratory rats, the BBB integrity indeed may be changed after pathogenic rabies virus infection.

Serial sampling of CSF from rabies virus-infected rats presents a dynamic change of the BBB permeability. We found that serum proteins, albumin and total IgG, were elevated on day 7 p.i. and increased further afterwards. Our preliminary study showed that the rabies virus could retrogradely replicate in the brain tissues on day 5 p.i. and clinical symptoms were observed on day 9 p.i.. We postulate that the initiation of the BBB opening was behind the replication of rabies virus in the CNS but prior to the appearance of clinical signs. Further, we also demonstrated that the disruption of BBB could allow macromolecular markers, e.g., albumin and IgG, to leak into the CNS. This result was substantiated by the Evans blue staining. The greater permeability was seen in the spinal cord and cerebellum compared with the cerebral cortex on day 10 to 11 p.i.. This similar regional difference was reported also in the attenuated CVS-F3 study in mice [27]. Together, these observations indicate that pathogenic rabies virus infection in the nervous tissues could allow the serum proteins passing through the BBB and loss of BBB integrity was more extensive to molecules from 66 to 150 kDa in the cerebellum and spinal cord than the cerebral cortex in rats.

Tight junction proteins, the essential components of the BBB, are the primary barrier to maintain the brain homeostasis [26]. Alterations of tight junction protein expression and discontinuous cell junctions have been demonstrated in several CNS diseases, including trauma, cerebral infarction, multiple sclerosis, viral infection, and neurodegeneration [28-31]. Our analysis of major tight junction proteins revealed a unique susceptibility of occludin but not ZO-1 after rabies virus infection in both the cerebral cortex and cerebellum. Decreased expression of occludin leads to increased BBB permeability [32-34]. These findings are consistent with our present study demonstrating that the decreased expression of occludin is accompanied by BBB hyperpermeability in rabies virus-infected rats. Our findings indicate that rabies virus infection could trigger the BBB disruption. Nevertheless, other tight junction proteins, such as claudins and junctional adhesion molecule also contribute structurally to the formation of BBB [34,35]. These proteins may participate in the rabies mediated changes of BBB permeability. However, more investigations are needed to clarify its role.

According to the results of quantitative ELISA and Evans blue study, the rabies virus infection induced BBB leakage of macromolecules was restricted to the cerebellum and spinal cord. We compared hypertonic arabinose, TNF-α, and FUS to find out which method could enhance greater BBB disruption and allow more macromolecules, such as IgG, to enter the brain. Intracarotid infusion of a hypertonic solution caused a transient and reversible shrinkage of the endothelial cells and opening of the endothelial tight junctions and increasing the delivery of antineoplastic agents, neutralizing antibodies, mAbs, nanoparticles and viral vectors into the CNS [36-40]. Intracarotid and intracerebral rTNF-α administration could result in BBB opening for different tracers, such as sodium fluorescein, horseradish peroxidase, albumin, and endogenous IgG [41,42]. FUS combined with microbubbles has been shown to be capable of BBB opening, and it improved the delivery of anti-tumor compounds and antibodies into the brain [43,44]. We found that FUS and hypertonic arabinose could effectively open the BBB and raise the concentrations of macromolecules detected in the CSF. In contrast, exogenous rTNF-α injection did not induce BBB leakage for large molecules and even inhibited BBB permeability. However, we could not exclude the possibility that small molecules may pass through the BBB.

Although BBB permeability was changed after rabies virus infection, the mortality was not altered. This is mainly resulted from the insufficient titer of neutralization antibody for rabies virus, which was substantiated by the finding of lower levels of virus-specific and neutralizating antibody in the sera and the CSF. This result is similar to human naturally infected with rabies virus. They do not receive PEP and generally do not mount a strong immune response to rabies relatively late in the disease [7,45]. VNA can be detected in the sera and CSF, but the titers are usually relatively low [7,12]. Insufficient antibodies could not provide appropriate protection in peripheral and central nervous tissues. It is surmised that enhancing the BBB permeability without a concomitant increase of antibodies in the CNS would be futile to protect against the lethality after rabies virus infection. In this regard, it is important to note that delivery of high levels of virus-neutralizing mAb to the CNS tissues should diminish lethality and clear rabies virus.

It has also been reported that exogenous neutralizing antibodies accompanied with the enhancement of BBB permeability can raise the survival more than the administration of monoclonal antibodies alone [46,47]. In our study, PEP treatment using virus-neutralizing mAb 8-10E combined with BBB disruption elevated the survival in LEW rats inoculated with rabies virus on day 7 p.i., however; the effect was diminished on day 9 p.i.. One possible explanation may be that the adverse effects of osmotic infusion, such as seizures, reinforce damage to the brain already exhibiting neuronal dysfunction [48]. It is surprising to note that neutralizing mAb 8-10E treatment alone increased the survival rate by 33.3% and 28.6%. respectively, on day 7 and 9 p.i. We hypothesize that administration of sufficient neutralizing mAbs into the CNS could rescue the rats from rabies-induced lethality and early treatment with mAb also increase survival rate.

Until now, besides the success of the Wisconsin’s patient treated with the Milwaukee Protocol, several others have employed similar treatment regimens with minimal beneficial outcomes [6,49-51]. Although a fraction of rabies virus-infected rats could be saved with passive neutralizing mAb treatment, whether greater improvement could be achieved with immune therapy needs more studies. In future development, a passively administrated mAb or such a humanized mAb combined with effective and global BBB breakdown may provide an alternative therapy for rescuing patients who have been diagnosed with rabies.

## Conclusions

The present study revealed that BBB permeability was altered after pathogenic rabies virus infection in rats. Sufficient virus-neutralizing antibody plays a major role in determining the survival from rabies virus infection. Our study indicates that enhancing BBB opening combined with delivering sufficient virus-neutralizing mAb to the brain may provide an effective treatment when the CNS infection has been established.

## Competing interests

The authors declare that they have no competing interests.

## Authors’ contributions

PHL performed the major experiments and wrote the manuscript. HHY and PTC were responsible for virus isolation and mAb production. PCC and HLL were for technical support of FUS study. MHW participated in data interpretation and manuscript improvement. LKC conceived the study, designed the experiments and analyzed the data. All authors read and approved the final manuscript.
